# Circulating trimethylamine N-oxide is correlated with high coronary artery atherosclerotic burden in individuals with newly diagnosed coronary heart disease

**DOI:** 10.1186/s12872-024-03937-5

**Published:** 2024-05-21

**Authors:** Minghui Bao, Haotong Li, Jianping Li

**Affiliations:** 1 Department of Cardiology, Peking University First Hospital, Peking University, Xi Shiku Street No. 8, Xicheng District, Beijing, 100034 China; 2 National Center for Cardiovascular Diseases, Fuwai Hospital, Chinese Academy of Medical Sciences and Peking Union Medical College, Beijing, China

**Keywords:** Trimethylamine N-oxide (TMAO), Coronary heart disease (CHD), SYNTAX score, Newly diagnosed

## Abstract

**Background:**

Trimethylamine N-oxide (TMAO) is a metabolite derived from the gut microbiota and has been reported to be correlated with cardiovascular diseases. Although TMAO is associated with the severity of coronary artery disease in subjects with coronary heart disease (CHD) history. However, the correlation between TMAO and the atherosclerotic burden in newly diagnosed cases of CHD is unknown.

**Methods:**

In this hospital-based study, we enrolled 429 individuals newly diagnosed with CHD undergoing coronary angiography. Plasma TMAO was assessed before coronary angiography. SYNTAX score was computed during coronary angiography to estimate the coronary artery atherosclerotic burden. Both linear and logistic regression analyses were conducted to explore the correlation between plasma TMAO levels and SYNTAX score in newly diagnosed CHD population.

**Results:**

The TMAO in patients with SYNTAX ≥ 33 and subjects with SYNTAX < 23 were 6.10 (interquartile range [IQR]: 3.53 to 9.15) µmol/L and 4.90 [IQR: 3.25 to 7.68] µmol/L, respectively. Linear regression adjusting for traditional risk factors showed TMAO level was positively correlated with SYNTAX score (β = 0.179; *p* = 0.006) in CHD population. When TMAO was added to models with traditional risk factors, the predictive value improved significantly, with the receiver operating characteristic curve (AUC) increased from 0.7312 to 0.7502 (*p* = 0.003). Stratified analysis showed that the correlations did not hold true for subjects who were non-smoker or with histories of diabetes. None of the stratifying factors significantly altered the correlation (all p for interaction < 0.05).

**Conclusions:**

We found a positive linear correlation between plasma TMAO and SYNTAX score among newly diagnosed CHD individuals in Chinese population.

**Supplementary Information:**

The online version contains supplementary material available at 10.1186/s12872-024-03937-5.

## Introduction

Coronary heart disease (CHD) stands as one of the leading causes of death worldwide with its prevalence on the rise in general population [1, 2]. However, the exact pathogenic mechanisms of CHD have not been fully elucidated. Prompt recognition and management of individuals at high risk of CHD will help to decrease cardiovascular morbidity and mortality [3]. Previous studies have reported that approximately 20-30% of patients present with multivessel coronary artery disease [4, 5]. It has been well documented that the presence of severe non-IRA lesions in patients may be responsible for recurrent angina, repeat revascularization, and a poorer prognosis. Thus, the early identification and treatment of hold significant clinical importance for physicians [6].

Despite significant efforts being made to mitigate traditional cardiovascular risk factors, increasing incidence of CHD incidence and related mortality continue to be observed in many countries. Studies have shown that conventional risk factors such as hypertension, diabetes mellitus, hyperlipidemia, smoking, age, sex, and body weight can explain only 30% of adverse outcomes related to CHD [7, 8]. Therefore, it is far from enough in enhancing the prognosis of CHD by merely controlling those traditional risk factors. Hence, identifying novel pathogenic risk factors related to CHD may promote public health and aid in disease prevention and risk assessment [9].

With the rapid advancement of gut metagenomics research in recent years, numerous studies have demonstrated that trimethylamine N-oxide (TMAO), a metabolite originating from the gut microbiota, can accelerate atherosclerosis and contribute to adverse cardiovascular outcomes [10–14]. Dietary carnitine and phosphotidyl choline generated from red meat, are converted to trimethylamine (TMA) by colonic microbes. TMA is then converted to TMAO [15]. Significant associations have been found between TMAO and myocardial infarction (MI) including ST-segment elevated myocardial infarction (STEMI), non-ST-segment elevation myocardial infarction (NSTEMI) and stable coronary artery disease [16, 17]. However, this population may largely represent individuals with long lasting myocardial ischemia and underwent long-term CHD secondary preventive drug therapy. Whether this relationship still exists among individuals newly diagnosed with CHD remains unexplored. To address this issue, this study investigates the relationship between plasma TMAO levels in newly diagnosed patients with CHD in Chinese population.

## Materials and methods

### Participants

This was a prospective observational single-center study in Peking University First Hospital, Beijing, China. We consecutively included adults with suspected CHD undergoing coronary angiography. Electronic medical records were utilized to collect demographics, lifestyle, medical history, and disease status information. We enrolled individuals aged ≥ 18 years with suspected CHD underwent coronary angiography. The exclusion criteria were: (1) Previous diagnosed CHD by coronary angiography; (2) previous percutaneous coronary intervention (PCI) or coronary artery bypass surgery (CABG); (3) without informed consent for sample collection. The definition of CHD was in accordance with the 2019 ESC guideline of CHD [18]. All subjects signed an informed consent form, and the study complied with medical ethics standards.

From January 1, 2019, to December 31, 2019, 1061 patients were included in the participants screening. According to the inclusion and exclusion criteria, 875 were eligible for this study. 18 subjects without blood sample were excluded. Further, 428 participants without CHD (the greatest coronary stenosis degree < 50%) were excluded. As a result, 429 newly diagnosed CHD cases we finally included in the statistical analysis, including 317 chronic coronary syndrome (CCS) cases and 112 acute coronary syndrome (ACS) cases (89 unstable angina pectoris (UA) cases, 19 NSTEMI cases, and 4 STEMI cases).

### Plasma TMAO measurement

Blood samples were collected from radial or femoral access before heparinization during angiography using vacutainer tubes containing EDTA. Then the blood sample was centrifuged and stored at -80 °C. API 3200 triple quadrupole mass spectrometer (AB SCIEX, USA) was used to measure plasma TMAO according to the manufacturer’s instructions [19].

### SYNTAX score measurement

The SYNTAX score was assessed by trained physicians who were blinded to the TMAO levels. The SYNTAX score was computed using the online SYNTAX score calculator version 2.28 (www.syntaxscore.com/calculator/syntaxscore/frameset.htm). Patients were divided into three groups according to the SYNTAX score: low SYNTAX score group (SYNTAX score < 23), intermediate SYNTAX score group (23 ≤ SYNTAX score < 33), and high SYNTAX score group (SYNTAX ≥ 33). During coronary angiography, the stenosis degree of each involved vessel was recorded, and the vessel displaying the most severe stenosis was included in the final statistical analysis as the severest degree of stenosis.

### Definitions of diseases

Hypertension was regarded as meeting one or more of the criteria: SBP ≥ 140 mmHg or DBP ≥ 90 mmHg or physician-diagnosed hypertension or using antihypertensive drugs. Diabetes mellitus was regarded as meeting one or more criteria: physician-diagnosed diabetes, taking antidiabetic drugs, using insulin, glycated hemoglobin level ≥ 6.5%, FBG level ≥ 7.0 mmol/L, or a 2 h glucose level ≥ 11.1 mmol/L after an oral glucose tolerance test. Hyperlipidemia was regarded as physician-diagnosed hyperlipidemia or using lipid-lowering drugs.

### Statistical analyses

Continuous variables were presented as mean ± standard deviation (SD) or median (IQR). Categorical variables were expressed as proportions. Due to the distribution of values for TMAO being strongly skewed, TMAO underwent log_2_ transformed. The correlation between TMAO and SYNTAX score was investigated using linear regression model as a continuous variable per 1 µmol/L increase in log_2_TMAO. Odds ratios (ORs) and 95% confidence intervals (95% CIs) for SYNTAX in relation to TMAO were calculated using logistic regression models. Two-tailed *p* < 0.05 was considered as statistically significant. R software (version: 4.2.2; http://www.R-project.org) was adopted for statistical analysis.

## Results

### Baseline characteristics of the study participants

**Fig. 1:**
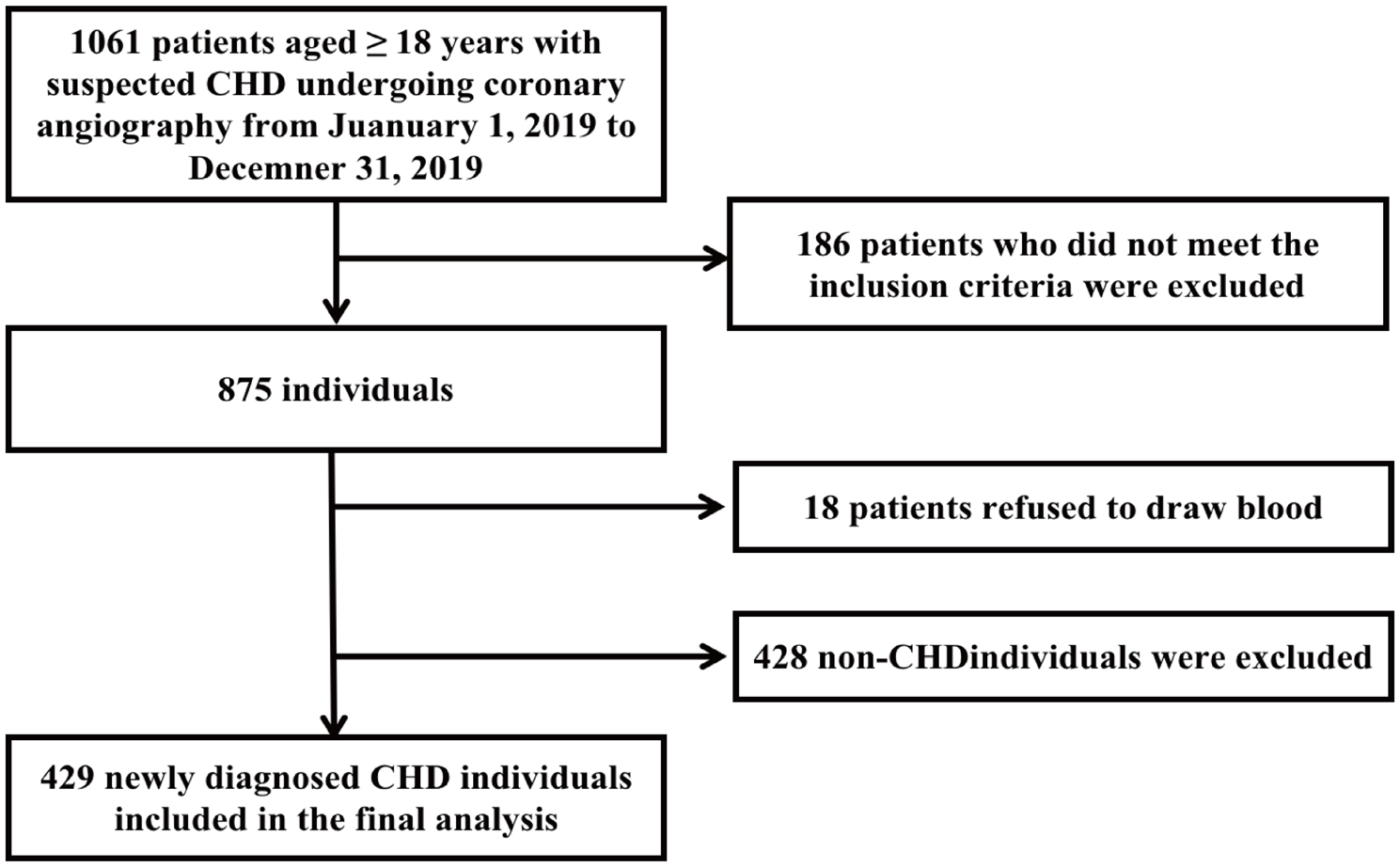
A flow chart of this study. CHD, coronary heart disease; ACS, acute coronary syndrome; CHD, chronic coronary syndrome

A total 429 newly identified CHD cases met the inclusion criteria were included in the final analysis (Fig. 1**)**. Patients were grouped into low, intermediate, and high atherosclerosis burden groups based on their SYNTAX scores. The mean age (SD) was 63.89 (10.48) years; with 46.9% being male; 72.7% were hypertensive patients; 48.1% had a diagnosis of diabetes mellitus; 71.1% of subjects were diagnosed with dyslipidemia; 46.5% subjects had a smoking history. The average syntax score was 17.44 (13.41) points and the median TMAO level was 5.90 µmol/L [IQR: 4.90 to 8.30]. Individuals with greater atherosclerotic burden are more likely to be male (44.3% vs. 67.5% in SYNTAX < 23 and SYNTAX ≥ 33 groups, respectively; *p* = 0.021), with higher SBP (132.28 mmHg vs. 137.13 mmHg in SYNTAX < 23 and SYNTAX ≥ 33 groups, respectively; *p* = 0.019), had a larger number of involved arteries (1.84 vs. 2.85 in SYNTAX < 23 and SYNTAX ≥ 33 groups; *p* < 0.001), and had a more severe coronary stenosis degree (87.65% vs. 96.47% in SYNTAX < 23 and SYNTAX ≥ 33 groups, respectively; *p* < 0.001) (Table 1). As the distribution of TMAO was skewed (Supplementary Fig. 1A), we therefore conducted log_2_ transformation for TMAO to enhance its suitability for further analysis (Supplementary Fig. 1B).

**Table 1:** Baseline characteristics of CHD individuals with different SYNTAX score

	Overall population *N* = 429	SYNTAX < 23 *N* = 314	23 ≤ SYNTAX < 33 *N* = 75	SYNTAX ≥ 33 *N* = 40	*P* value
Female (%)	228 (53.1)	175 (55.7)	40 (53.3)	13 (32.5)	0.021
Age (year)	63.89 (10.48)	63.25 (10.59)	66.44 (9.10)	64.15 (11.48)	0.059
SBP (mmHg)	133.65 (16.87)	132.28 (15.28)	137.67 (19.11)	137.13 (22.58)	0.019
DBP (mmHg)	74.23 (10.68)	74.11 (10.79)	74.44 (10.42)	74.85 (10.57)	0.906
BMI (kg/m^2^)	25.94 (3.65)	26.05 (3.80)	25.59 (3.49)	25.77 (2.62)	0.599
Hypertension (%)	309 (72.7)	219 (80.4)	61 (81.3)	29 (74.4)	0.158
Diabetes mellitus (%)	205 (48.1)	145 (46.5)	44 (58.7)	16 (41.0)	0.107
Dyslipidemia (%)	298 (71.1)	212 (69.3)	59 (78.7)	27 (71.1)	0.275
Smoking history (%)	197 (46.5)	139 (44.7)	35 (47.3)	23 (59.0)	0.239
Drinking history (%)	128 (30.4)	90 (29.0)	23 (31.1)	15 (40.5)	0.352
CHD family history (%)	154 (38.2)	115 (38.6)	26 (38.2)	13 (35.1)	0.92
Hcy (mmol/L)	14.75 (9.84)	14.22 (7.90)	15.84 (15.00)	16.69 (10.36)	0.297
LDL-C (mmol/L)	2.28 (0.81)	2.30 (0.81)	2.21 (0.80)	2.26 (0.81)	0.672
SYNTAX score (n)	17.44 (13.41)	11.32 (5.42)	27.12 (2.79)	47.42 (17.82)	< 0.001
Number of arteries (n)	2.08 (0.82)	1.84 (0.78)	2.65 (0.58)	2.85 (0.49)	< 0.001
Stenosis degree (%)	89.59 (8.88)	87.65 (8.87)	94.05 (6.68)	96.47 (5.56)	< 0.001
TMAO (µmol/L)	5.90 [4.90, 8.30]	4.90 [3.25, 7.68]	5.10 [4.73, 8.98]	6.10 [5.35, 9.15]	0.018
log_2_TMAO (µmol/L)	2.56 (1.27)	2.29 (1.15)	2.35 (1.30)	2.61 (1.20)	0.012

SBP, systolic blood pressure; DBP, diastolic blood pressure; BMI, body mass index; CHD, coronary heart disease; LDL-C, low density lipoprotein cholesterol; TMAO, trimethylamine N-oxide

### Association between plasma TMAO and coronary atherosclerotic burden

Plasma log_2_TMAO levels were significantly correlated with the SYNTAX score in newly diagnosed CHD patients (Pearson’s correlation coefficient: *r* = 0.439, *p* < 0.001). Based on the linear regression model, in model 1, we treated SYNTAX score as dependent variable and _log2_TMAO as independent variable. The results showed a positive correlation between log_2_TMAO and SYNTAX score. For each 1 µmol/L increase in log_2_TMAO there was associated with a 0.287 SD increase in the SYNTAX score (*p* = 0.001). In model 2, we used SYNTAX score as the dependent variable and including log_2_TMAO, age, BMI, SBP, DBP, smoking status, drinking status, self-reported hypertension, self-reported diabetes, self-reported hyperlipidemia, family history of CHD, LDL-C and Hcy as independent variables. Significant correlation was also identified in this multivariable adjusted model (β = 179, *p* = 0.006). Additionally, when log_2_TMAO was evaluated in quantiles, significantly higher SYNTAX score was identified in the third (Model 1: β = 0.241, *p* < 0.001; Model 2: β = 0.229, *p* < 0.001) and the fourth (Model 1: β = 0.433, *p* < 0.001; Model 2: β = 0.412, *p* < 0.001) quantiles in both unadjusted and adjusted models. See Table 2 for detailed information.

**Table 2:** Association between TMAO and coronary atherosclerotic burden in CHD individuals

Log_2_TMAO µmol/L	*N*	Model 1				Model 2			
		β	t	*p* value	95% CI	β	t	*p* value	95% CI
Per 1 µmol/L increase	429	0.287	2.916	0.001	0.173 ~ 0.420	0.179	1.832	0.006	0.096 ~ 0.308
**Quantiles**									
Q1	108	0.022	0.288	0.721	-0.168 ~ 0.283	0.016	0.173	0.819	-0.168 ~ 0.283
Q2	108	0.067	0.684	0.076	-0.079 ~ 0.303	0.059	0.638	0.298	-0.071 ~ 0.219
Q3	106	0.241	2.589	< 0.001	0.112 ~ 0.431	0.229	2.381	< 0.001	0.082 ~ 0.391
Q4	107	0.433	4.607	< 0.001	0.139 ~ 0.728	0.412	4.207	< 0.001	0.104 ~ 0.688

Model 1, unadjusted model; model 2, adjusted for traditional risk factor including age, sex, BMI, SBP, DBP, smoking status, drinking status, self-reported hypertension, self-reported diabetes, self-reported hyperlipidemia, family history of CHD, LDL-C and Hcy; SBP, systolic blood pressure; DBP, diastolic blood pressure; BMI, body mass index; CHD, coronary heart disease; LDL-C, low density lipoprotein cholesterol; TMAO, trimethylamine N-oxide; OR, odds ratio; Cl, confidence interval; Q1, the first quantile of log_2_TMAO; Q2, the second quantile of log_2_TMAO; Q3, the third quantile of log_2_TMAO; Q4, the fourth quantile of log_2_TMAO

### Stratified analysis for plasma TMAO and intermediate to high coronary atherosclerotic burden

**Fig. 2:**
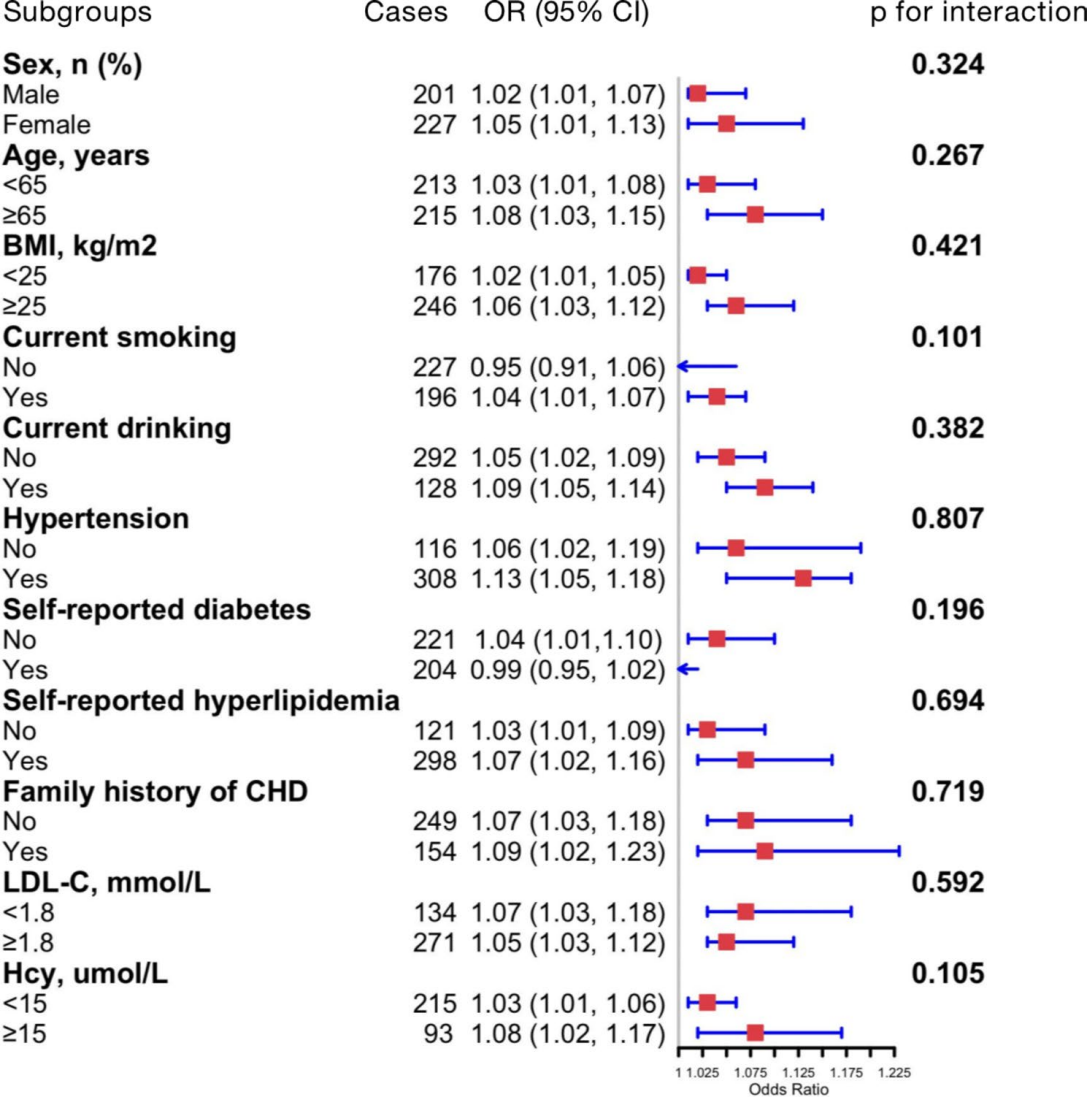
Subgroup and interaction analyses for the association between log_2_TMAO and intermediate to high coronary atherosclerotic burden of CHD patients. Each subgroup analysis adjusted, if not stratified, adjusted for age, BMI, smoking status, drinking status, hypertension, diabetes, hyperlipidemia, family history of CHD, LDL-C, and Hcy. CHD, chronic coronary syndrome; SBP, systolic blood pressure; DBP, diastolic blood pressure; BMI, body mass index; CHD, coronary heart disease; LDL-C, low density lipoprotein cholesterol; TMAO, trimethylamine N-oxide; OR, odds ratio; Cl, confidence interval

Given the significant positive correlation between TMAO and SYNTAX, stratified analysis was conducted to explore whether the correlation remains significant in different subgroups. Individuals with SYNTAX ≥ 23 were classified as subjects with intermediate to high coronary atherosclerotic burden, while individuals with SYNTAX < 23 were regarded as subjects with low burden. The results indicated that most subgroups exhibited positive correlations between TMAO and SYNTAX score. However, subjects who were non-smoker, and having diabetes mellitus failed to show significant correlation. None of these variables were observed to alter the correlation between TMAO and atherosclerotic burden (all p for interaction > 0.05) (Table 3; Fig. 2).

**Table 3:** Stratified analysis for the correlation between TMAO and intermediate to high coronary atherosclerotic burden in CHD individuals

Subgroups	*N*	OR (95% CI)	*p* for interaction
**Sex, n (%)**			0.324
Male	201	1.02 (1.01, 1.07)	
Female	227	1.05 (1.01, 1.13)	
**Age, years**			0.267
< 65	213	1.03 (1.01, 1.08)	
≥ 65	215	1.08 (1.03, 1.15)	
**BMI, kg/m** ^**2**^			0.421
< 25	176	1.02 (1.01, 1.05)	
≥ 25	246	1.06 (1.03, 1.12)	
**Current smoking**			0.101
No	227	0.95 (0.91, 1.06)	
Yes	196	1.04 (1.01, 1.07)	
**Current drinking**			0.382
No	292	1.05 (1.02, 1.09)	
Yes	128	1.09 (1.05, 1.14)	
**Hypertension**			0.807
No	116	1.06 (1.02, 1.19)	
Yes	308	1.13 (1.05, 1.18)	
**Self-reported diabetes**			0.196
No	221	1.04 (1.01,1.10)	
Yes	204	0.99 (0.95, 1.02)	
**Self-reported hyperlipidemia**			0.694
No	121	1.03 (1.01, 1.09)	
Yes	298	1.07 (1.02, 1.16)	
**Family history of CHD**			0.719
No	249	1.07 (1.03, 1.18)	
Yes	154	1.09 (1.02, 1.23)	
**LDL-C, mmol/L**			0.592
< 1.8	134	1.07 (1.03, 1.18)	
≥ 1.8	271	1.05 (1.03, 1.12)	
**Hcy, µmol/L**			0.105
< 15	215	1.03 (1.01, 1.06)	
≥ 15	93	1.08 (1.02, 1.17)	

SBP, systolic blood pressure; DBP, diastolic blood pressure; BMI, body mass index; CHD, coronary heart disease; LDL-C, low density lipoprotein cholesterol; TMAO, trimethylamine N-oxide; OR, odds ratio; Cl, confidence interval

### TMAO for predicting high coronary atherosclerotic burden

**Fig. 3:**
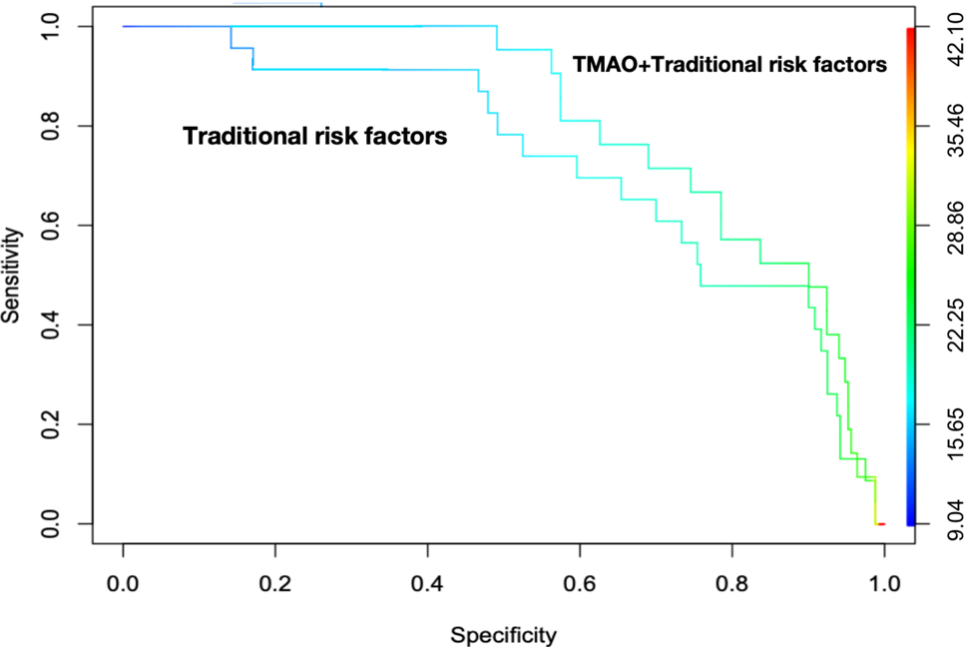
ROC curves for predicting intermediate to high coronary atherosclerotic burden of CHD patients. ROC curve of traditional cardiovascular risk factors including age, BMI, smoking status, drinking status, hypertension, diabetes, hyperlipidemia, family history of CHD, LDL-C, and Hcy and ROC curve of the combination of log2TMAO and traditional cardiovascular risk factors. AUC, area under the receiver-operating characteristic curve

The discrimination abilities of a model incorporating traditional cardiovascular risk factors, such as BMI, age, SBP, DBP, smoking, drinking, hypertension history, diabetes mellitus history, CHD history, dyslipidemia history, LDL-C, and Hcy, were initially estimated using ROC curves and AUC. The results showed that the AUC of the combination of traditional risk factors was 0.7312. Subsequently, we added the log_2_TMAO to the traditional model to construct a new model. The AUC of the new model increased to 0.7502 (Fig. 3). Model comparison revealed the discrimination ability of the new model outperformed the traditional model. The difference between models reaching statistical significance (*p* = 0.0027).

## Discussion

This study is the first to explore the relationship between plasma TMAO and coronary atherosclerotic burden among individuals who were newly diagnosed with CHD. The notable finding of this study was a significant association between plasma TMAO levels and intermediate to high atherosclerotic burden represented by high SYNTAX score. The correlation remains significant even following adjusting for traditional cardiovascular risk factors.

Atherosclerosis is one of the most significant clinical concerns resulting in stenosis and occlusion within the artery system [18, 20]. Once involved in the coronary vessels, partial or total vessel occlusion can lead to gradual reduction or complete block of coronary blood flow [21, 22]. Patients suffered from this condition may present as asymptomatic disease, angina pectoris, or myocardial infarction, and finally result in cardiovascular events such as heart failure, arrhythmias, and death [23]. Therefore, there is an urgent need to develop effective risk prediction models to identify individuals with high atherosclerotic burdens and adverse outcomes.

The gut microbiota contributes to various cardiovascular diseases such as coronary atherosclerosis, hypertension, and heart failure [24, 25]. Choline is a trimethylamine-containing compound is metabolized by the gut microbiota to produce trimethylamine. Further, trimethylamine is oxidized by hepatic flavin monooxygenase 3 to generate TMAO [26]. Animal experiments showed that, dietary supplementation with TMAO in hyperlipidemic mice can promote atherosclerosis, suggesting TMAO is involved in atherosclerosis and the development of CVD [10, 15]. TMAO promotes atherosclerosis through inhibiting cholesterol reverse transport, enhancing platelet activity, and promoting thrombosis. However, interfering with these processes will attenuate pathogenesis of atherosclerosis [11, 15, 27, 28]. Clinical studies showed a close relationship of TMAO with the occurrence of adverse cardiovascular outcomes [12, 29]. Several human studies have identified that circulating TMAO level is an independent predictor of multivessel disease with STEMI. A recent study showed that sustained high TMAO level were responsible for higher CVD risk. Consequently, repeated measurement of TMAO may promote the early identification of subjects with greater CVD risk [30]. Mechanistically, choline or TMAO supplementation may promote the formation of foam cells by increasing the number of macrophages scavenger receptors [10]. Consequently, TMAO impairs the balance in cholesterol uptake and efflux, leading to great number of foam cells migrating into the arterial wall. Another pathogenic mechanism of TMAO is inflammation [31]. TMAO is correlated with inflammation by promoting inflammatory factor expression, such as TNF-α [32] and C-reactive protein [33].

The findings of this study have some clinical implications. Data from the present study indicate a positive correlation between plasma TMAO concentration and the severity of coronary artery lesions among newly diagnosed patients with CHD. The relationship persists after adjusting for several traditional risk factors. Hence, our findings suggest that TMAO may potentially facilitate the identification of CHD individuals with high atherosclerosis burden. This study has several limitations. First, this was a single-center study. Whether the results of this study can be generalized to other populations need validation. Second, we did not collect the information about patients’ nutritional status and recent diet. Third, we are unable to establish a causal relationship due to the constraints of the available evidence.

## Conclusions

In summary, this study reveals a positive correlation between plasma TMAO and high atherosclerotic burden in newly diagnosed CHD subjects among Chinese population. Our results may aid in identifying individuals with elevated atherosclerotic burden.

### Electronic supplementary material

Below is the link to the electronic supplementary material.

Supplementary Material 1

## Data Availability

The data that support the findings of this study are available from the corresponding author on reasonable request.
