# Supplementary material for: Circulating trimethylamine N-oxide is correlated with high coronary artery atherosclerotic burden in individuals with newly diagnosed coronary heart disease
Source: BMC Cardiovasc Disord. 2024 May 21;24:265. doi: 10.1186/s12872-024-03937-5 (PMC11106919; doi:10.1186/s12872-024-03937-5)
Supplement: Supplementary file 1 — Supplementary Material 1 [file 12872_2024_3937_MOESM1_ESM.docx]

**
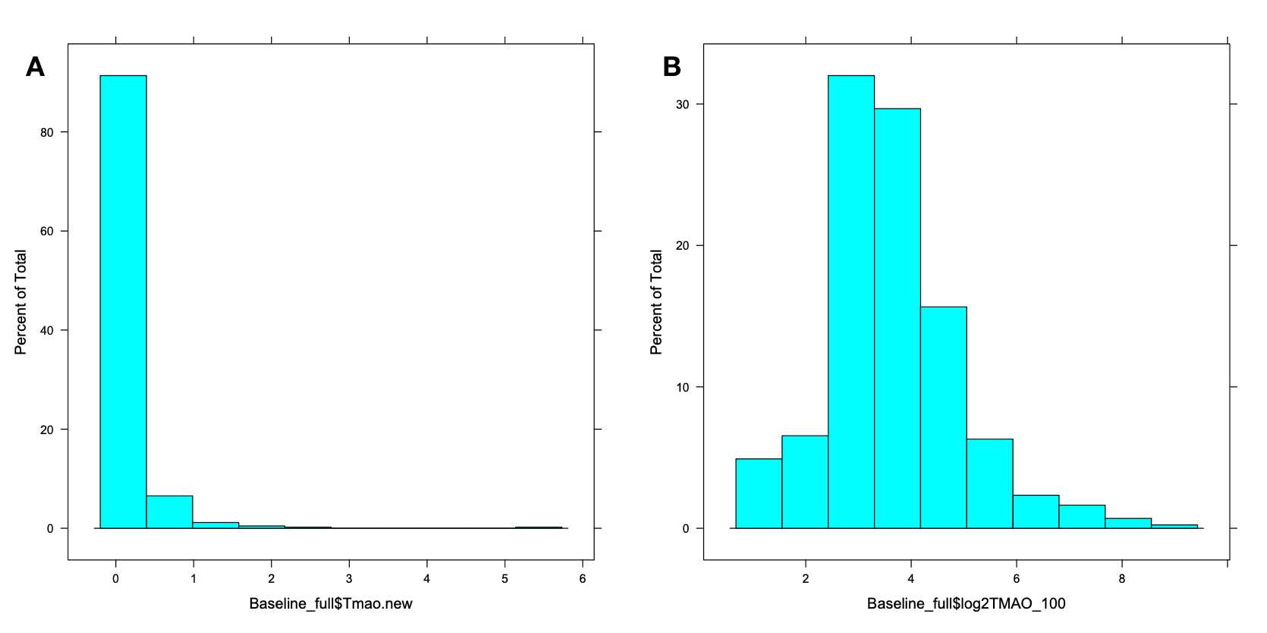
**

**Supplementary Figure 1. Distributions of plasma TMAO in CHD individuals.** (A) plasma TMAO levels; (B) log_2_ transformation for plasma TMAO levels.
